# Stochastic colonization, transient and boom–bust dynamics shape invasions by native taxa

**DOI:** 10.1002/ecy.70398

**Published:** 2026-05-06

**Authors:** Daniel Oro, Giulio Tirabassi

**Affiliations:** ^1^ Ecology and Complexity Group Centre d'Estudis Avançats de Blanes CEAB (CSIC) Blanes Spain; ^2^ Department of Computer Science, Applied Mathematics and Statistics Universitat de Girona Girona Spain; ^3^ Department of Physics Universitat Politècnica de Catalunya Terrassa Spain

**Keywords:** climate, ecological succession, environmental stochasticity, functional groups, invasion dynamics, invasion success, life histories, long transient dynamics, open systems, spatial heterogeneity

## Abstract

Boom–bust dynamics (BBD) is a population dynamic pattern described in invasion ecology, where a population suddenly increases (boom) before quickly declining (bust), eventually stabilizing at much lower levels. This initial outbreak is considered a transient phase preceding a long‐term population equilibrium. If BBD is common in invasions, understanding it is crucial for invasion interpretations and management. Nevertheless, how frequently BBD occurs is little known due to a scarcity and quality of data, and its underlying drivers remain poorly understood, especially among native species recolonizing restored habitats. Restored ecosystems offer a rare opportunity to investigate BBD in the absence of non‐native traits. Here, we analyzed BBD in a community of 47 breeding waterbirds across 53 wetland patches from 1984 to 2023, where restoration measures (e.g., regulated hunting, improved hydrology) triggered an invasion (recolonization) process. Strikingly, although the study spanned 3–8 generation times, most species and the whole community showed long transient dynamics far from equilibrium. BBD was observed in over 75% of cases, exhibiting single and recurring patterns, particularly in wetlands with stochastic hydrology and among generalist species. Patch features influenced the occurrence of BBD nonlinearly, likely through interactions with other features, especially with species ecological typology. Rare species showed particular dynamics, characterized by high colonization–extinction turnover. Our findings, although restricted to waterbirds, suggest that, at least in this dataset, BBD are prevalent during invasions. Moreover, only approximately 20% of colonizations achieved long‐term persistence, while the majority resulted in local extinctions. This underscores that failed, often undetected, colonization attempts may be widespread. Importantly, our study also shows that BBD can emerge in native species through intrinsic ecological processes alone, without invoking exotic traits or the species' ecological novelty. This challenges the prevailing view that BBD are uniquely associated with alien species and calls for a reinterpretation of population dynamics after colonization.

## INTRODUCTION

Several patterns of population dynamics have been described during the colonization or recolonization of a patch, such as logistic, exponential, lagged‐time, linear, biphasic, and boom and bust, depending on environmental conditions and the life history demographic traits of the species arriving (Krebs, 2009; Turchin, 2003). How populations colonize new areas and fluctuate over time has been widely debated, particularly regarding the frequency and features of “boom–bust” dynamics (BBD hereafter), which have been described for biological invasions. This term describes a pattern after invasion in which populations rapidly increase to high densities (“boom”) and subsequently experience a sharp decline or even a crash (“bust”) (Strayer et al., 2017). BBD include single or multiple recurrent patterns, as well as highly stochastic and noisy dynamics after colonization. Almost a hundred years ago, Charles Elton already noted that “many of the most striking cases of sudden increase in animals occur when a species is introduced into a country strange to it, in which it does not at first fit harmoniously” (Elton, 1927). Elton also detailed a well‐known failed invasion caused by the collapse of a largely abundant invasive species, the Canadian pondweed (Elton, 1958). Since then, the ecology of invasions and their ecological consequences for ecosystem functioning have attracted much scientific attention (Davis, 2009; Hui & Richardson, 2017; Lockwood et al., 2013). Nevertheless, challenges in empirical data and quantitative approaches to studying invasive species dynamics persist. For instance, population dynamics of invasive species can be far from a logistic pattern (from the initial slow density increase to exponential growth and a final stabilization), and the population boom may correspond to a long transient phenomenon that has been little explored in this context (Hastings et al., 2018; Morozov et al., 2020). While most literature on BBD focuses on alien and exotic species (Strayer et al., 2017), invasion processes can also involve native species experiencing range expansions (Cunze & Klimpel, 2022; Payo‐Payo et al., 2018; Rocha‐Camarero & de Trucios, 2002). For these later species, there is also limited knowledge of BBD during colonization and recolonization processes (Turchin, 2003).

Although some extensive reviews on invasion ecology suggest that BBD would be relatively rare for organisms invading a new habitat (Simberloff & Gibbons, 2004; Williamson, 1996), others state that it would be rather common (Davis, 2009; Duncan et al., 2020; Hui & Richardson, 2017; Lockwood et al., 2013). Strayer et al. (2017) reviewed BBD in ecological invasions and concluded that we urgently require more empirical analyses of long‐term datasets for exploring their occurrence relative to other population dynamics, and a deeper understanding of the drivers and mechanisms influencing interactions between invasive species and their environments. This knowledge gap has also been limited by a lack of quantification and a suitable definition of the criteria used to categorize a set of time series (Strayer et al., 2017). A proper quantitative approach should reliably quantify how frequent BBD are after invasions, a central debate in invasion ecology. The current literature reveals a scarcity of empirical studies, which are often geographically limited, habitat‐specific, taxonomically narrow, and lacking in rigorous quantitative analysis. Furthermore, the temporal window for studying invasions is often too short to capture transient phenomena before populations reach an asymptotic regime (Hastings et al., 2018; Stott et al., 2010). An additional challenge, little considered when dealing with the processes of invasions and colonizations, is to record those that fail to establish viable populations, either because colonizers remain few or go extinct, and the process remains unnoticed (e.g., small initial propagule size causing Allee effects and demographic stochasticity) (Schreiber & Lloyd‐Smith, 2009; Simberloff, 2009).

Studying the invasion process for a range of organisms over sufficient time to attain a dynamic equilibrium or to go extinct has fundamental implications for understanding how populations invading a patch respond to the new environment (both biotic and abiotic). Here, we analyze the occurrence of BBD in a community of 47 breeding waterbirds across 53 Mediterranean patches (coastal wetlands) over 40 years, based on a uniquely spatially and temporally complete dataset where the restoration of depauperate habitats triggered a recolonization process. These wetlands were heavily and historically altered by anthropogenic impacts, for example, partial desiccation, fishing and hunting (Marco‐Barba et al., 2019; Soria, 2006; Vera et al., 2024). More recently, but still before protection, pollution from intensive agriculture and aquifer overexploitation further reduced habitat heterogeneity and suitability, leaving very low densities of only a few generalist waterbirds (Martínez‐Abraín & Giménez, 1987). Thus, recolonization occurred in nearly empty patches, where conditions improved abruptly (e.g., regulating harvesting, uncontrolled pollutant spills) or gradually (e.g., improving water quality). We considered the recolonization as functionally similar to an invasion, that is, species should interact with novel local biotic and abiotic conditions, generating strong transient dynamics (Iles et al., 2016). This similarity would occur at the patch scale, where establishment and early fast growth would occur in newly suitable habitat following depauperation, whereas native recolonization and alien invasion would not likely share similar regional context, propagule sources, or landscape‐level population structure (Brown & Barney, 2021; Simberloff, 2009). In this context, we use invasion theory as a conceptual framework to study early population dynamics in restored systems, which remains informative even if differences between recolonization by native species and alien invasions may occur (e.g., coevolutionary history, enemy release). Most species in our dataset are likely recolonizing sites within their historical regional ranges such that dynamics largely reflect local re‐expansions from nearby source populations in central and northern Europe, where environmental conditions remained more favorable and stable, rather than true biogeographic range shifts (Yésou et al., 2012). Following proposed drivers and mechanisms of BBD and related transient dynamics (Hastings et al., 2018; Stott et al., 2010; Strayer et al., 2017), we tested whether species ecological typology, generation time (as a proxy of life history pace), diet, preferred habitat and conservation status (a proxy of regional abundance) influenced the occurrence of BBD relative to other shapes of population dynamics. We predicted that BBD would be more frequent in species with shorter generation times (Duncan et al., 2020; Papacostas et al., 2017). We also predicted that rainfall, which drives most environmental stochasticity in Mediterranean coastal wetlands (Ruhí et al., 2012), was negatively associated with the occurrence of BBD, since higher water levels increase food availability and predator isolation, at least for most of the species. Finally, smaller patches (i.e., with fewer resources) should exhibit more BBD than larger ones (Turchin, 2003). Taken together, our analyses may shed light on the drivers generating BBD in the reassembling waterbird community.

## METHODS

### Data

**FIGURE 1:**
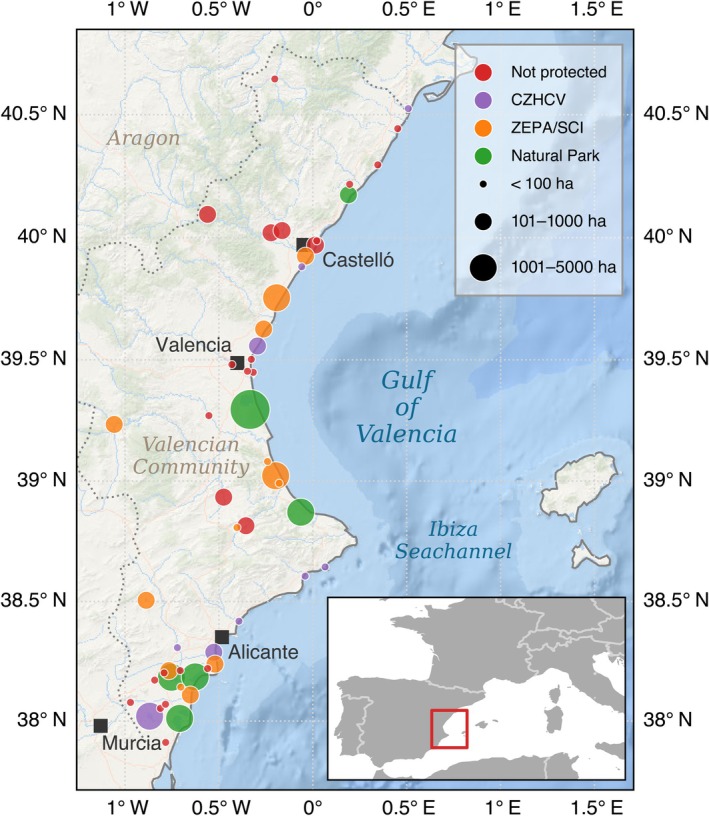
Geographical distributions, size, and protection status of the 53 patches where the sampling was carried out in this study.

The main data set we analyzed consists of counts of breeding pairs for a period of 40 years (1984–2023), compiled by the regional environmental authority in the western Mediterranean (Spain, Figure 1) (Martínez‐Abraín et al., 2016; Pagel et al., 2014). Breeding season counts were performed by the technical staff who monitored the study wetlands. Visits were performed almost daily throughout the whole breeding season (March–August) to prevent overlooking species due to differences in phenology. Counts were performed using specific methodologies for each species. Colonial species (herons, gulls, terns, shorebirds, and flamingos) were counted by visiting breeding colonies and counting individual nests at the peak of their incubation period. Non‐colonial species (ducks, coots, Podicipedidae) were detected by inspecting water masses using motor boats, counting nests or birds displaying breeding behavior, or adults with chicks. Abundances of species that are difficult to detect (e.g., grebes, little bittern) were estimated prospecting the study area in detail using boats propelled manually in shallow water areas (Pagel et al., 2014). We assumed that species detection probabilities can be considered constant across patches and mating seasons from year to year, as both methodology and human team composition have remained constant during the study period. However, we acknowledge that some unquantifiable bias may exist in such a type of monitoring (e.g., detectability depending on abundance, vegetation type, and life history traits—social vs. territorial, interannual differences in phenology).

**FIGURE 2:**
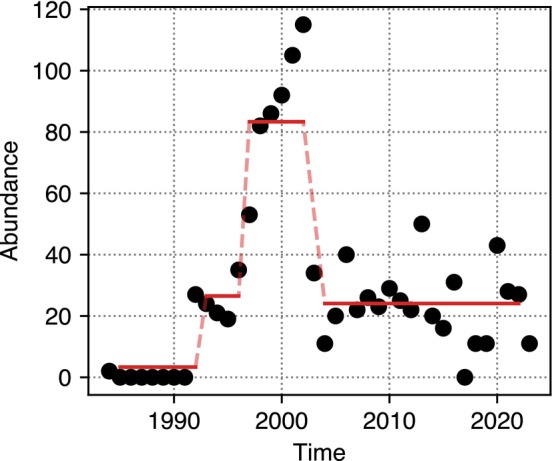
Example of regime shift detection for the time series of common coot (*Fulica atra*) in the patch Marjal de Almenara. The time series is reported as black dots. The red solid lines represent the average of the detected regimes. The time series presents three regime shifts between four different regimes, and it has been classified as Canonical boom‐bust.

Overall, 47 species were recorded and grouped by diet (specialists or generalists), habitat (freshwater or saltwater), conservation status (from lowest to highest concern: Least Concern, Near Threatened, Vulnerable, Endangered, and Critically Endangered), body mass, and ecological typology (ducks, rails, larosterna, waders, herons, pelecaniformes, grebes, flamingos, and raptors) (Appendix S1: Table S1). Figure 2 shows the temporal dynamics of species richness (a) and aggregated counts by ecological typology across patches (b). We also considered the generation time for each species (Bird et al., 2020) (median value: 5.52 years; range: 2.50–15.60 years).

We monitored 53 patches in the study area (median surface area: 80 ha; range: 1–21,008 ha; Figure 1). Most were former Holocene coastal lagoons heavily altered in their animal and plant communities (Martínez‐Abraín et al., 2016). Patches exceeding a minimum conservation threshold (i.e., hosting breeding populations of species of conservation concern) have been effectively protected since 1986–1988 as natural parks, the Catalogue of Wetlands of the Valencian Community (CZHCV, a regional statutory designation that provides legal recognition and land‐use protection), and Natura 2000 network (EU‐wide protected areas with binding obligations to prevent habitat deterioration and ensure favorable conservation status), either as Special Protection Areas for Birds (ZEPA/SPA) or as Site of Community Importance (SCI) (Pagel et al., 2014) (Figure 1). Overall, we have 640 abundance time series available, less than the maximum value of 2491 if all species were present in all patches.

Finally, most of the patches under analysis show a strong response to precipitation, which is in turn highly stochastic, as is typical of Mediterranean coastal areas. This variability has a significant impact on food availability for the species under examination, which is critically linked not only to water quality but also to water level. To test whether rainfall stochasticity affects population dynamics, we considered January–March cumulative rainfall per patch (ECMWF E‐OBS data31) (Cornes et al., 2018)—that is, previous to the breeding season. In particular, we aimed to assess whether anomalously high rainfall or drought conditions might trigger boom or bust phases, respectively. We stress, however, that we do not expect precipitation to influence all patches under analysis. Some patches are associated with human infrastructure (e.g., harbors), where water levels are actively managed and remain stable throughout the year.

### BBD detection

Despite the obvious limitations in ecological contexts, which are usually characterized by short and noisy time series, statistical inference has been used in the past to detect abrupt changes marking regime shifts in time series of population abundances (Andersen et al., 2009). In particular, methods such as the sequential *t* test analysis of regime shifts (STARS) (Rodionov, 2004; Rodionov & Overland, 2005) have been successfully used to detect shifts of productivity in marine fish stocks (Vert‐Pre et al., 2013), breakpoints in freshwater systems marking critical transitions (Gsell et al., 2016), and more recently it has been applied to unveil the connection between climatic regime shifts and a decline of the right whale population in the North Atlantic (Meyer‐Gutbrod et al., 2021). As pointed out in Strayer et al. (2017), such a method can be adapted to describe and detect particular dynamics, such as boom‐bust. To check its viability, the method was tested on synthetic data (Strayer et al., 2017) to verify if it was able to detect complicated regime shifts such as those associated with BBD. The authors found that this method could identify the boom‐bust as two regime shifts, producing fewer false positives for severe busts, less noisy data, and longer time series, and generally performed satisfactorily, better than other methods of regime shift detection. From this perspective, the STARS method was also employed in real datasets, in particular, to detect BBD in introduced crayfish populations (Sandström et al., 2014). The STARS method can be summarized as follows. Given a time series of abundances $x=\left\{x_{0},x_{1},\ldots ,x_{N}\right\}$, a sequential test is performed to check whether l consecutive points represent a significant anomaly with respect to the previous ones. In particular, given a regime that has a mean $x_{i}^{*}$ calculated from previous points up to i, the value that a new sample should exceed to represent an anomaly with a confidence level of p is computed as $x_{i}^{*}={\bar{x}}_{i}+\sqrt{\frac{2\sigma_{l}^{2}}{l}}$, where $\sigma_{l}$ is the average SD of all the l consecutive time series values and t is the value of a Student's *t* distribution with 2l−2 df at the given a priori Type I error rate α. Then, the following regime shift index is computed (Rodionov & Overland, 2005):

$$\mathrm{RS}I_{i}^{m}=\sum_{j=0}^{m}\frac{x_{i+j}-x_{i}^{*}}{l\;\sigma_{l}}.$$

If $\mathrm{RS}I_{i}^{m}>0$ for every m=0,…,l−1, then $x_{i}$ marks the beginning of a new regime, and the algorithm goes on, testing new points against this new regime. If the test fails, instead, $x_{i}$ is added to the current regime. This procedure would test for a new regime having a higher mean than the previous one. To test for regimes with a lower average, it is sufficient to consider $x_{i}^{*}={\bar{x}}_{i}-\sqrt{\frac{2\sigma_{l}^{2}}{l}}$ together with negative RSI values. By construction, the first regime is represented at least by the first l points of the time series, so if the regime shift happened earlier than l time steps, the method would not detect it. However, after inspecting the dataset under analysis, we believe this limitation does not affect our results, since we will employ a small value for l, which translates into only a small possible error, limited to the year of the first regime change.

The hyperparameter l should be tailored accordingly to the problem and the time series under examination. In particular, when the cutoff length is reduced, the time scale of regimes detected becomes shorter (Rodionov & Overland, 2005). We decided to use one value for all the time series, choosing l=6 years, as it represents the value with the lowest sensitivity producing meaningful regimes. The a priori Type I error rate α was set at 1%. To avoid the identification of 1‐year regimes, which are artifacts returned by the algorithm, a running mean of 3 years was applied to the time series.

Once a time series is reduced into a set of n regimes having average abundances of ${\bar{x}}_{k}$ with $k=\left\{1,\ldots ,n\right\}$, they are classified into the following dynamics classes:

1. *Single boom‐bust*: if the ${\bar{x}}_{k}$ have a single relative maximum
2. *Multiple boom‐busts*: if the ${\bar{x}}_{k}$ have multiple relative maxima separated by relative minima
3. *Rise*: if ${\bar{x}}_{1}<{\bar{x}}_{2}<\ldots <{\bar{x}}_{n}$
4. *Decline*: if ${\bar{x}}_{1}>{\bar{x}}_{2}>\ldots >{\bar{x}}_{n}$

Time series not falling into these classes are classified as Other. Such a definition of boom‐bust is quite broad and, depending on the time series, it might depart from the ideal scenario of a sudden growth from a relatively low baseline followed by an abrupt collapse, stabilizing the time series above the previous baseline, but still far from the peak. Therefore, we will also consider a subclass of the *Single boom–bust* and *Multiple boom–busts* classes, characterized by (1) a peak regime lasting more than a year and having an average of more than 50 counts per year, (2) a baseline regime having on average less than 10 counts per year, and (3) a final regime having an average count less than 50% of the peak regime. We will name this subclass *Canonical boom‐bust*. An example of this pattern is depicted in Figure 2. Given that the STAR algorithm is based on a Student's *t* distribution, regime detection in population time series displaying a high variance relative to the mean is particularly difficult. In particular, time series representing intermittent colonization attempts by a few individuals will be reduced to a high number of spurious regimes. Therefore, we will apply this algorithm only to the time series that display a relatively high average count across the monitoring period.

### BBD statistical analysis

To examine the relationship between the time series dynamics class and the associated metadata, we conducted χ^2^ tests for categorical variables (patch, habitat, diet, conservation status, and typology) and a Kruskal–Wallis *H* test for the continuous, non‐Gaussian variables of body mass and generation times.

To uncover possible multivariate influences of the time series metadata on the dynamics, we classified *Single boom‐bust* regimes using a gradient boosting algorithm, using as predictors patch, typology, status, habitat, diet, mass, and generation time. For each feature, the algorithm provides a measure of how much it contributes to reducing error in the model's predictions, called feature importance. To prevent overfitting, the model has been trained on 70% of the data (71 data points), leaving the rest for validation. Validation has been carried out based on model accuracy (fraction of correct classifications) and F1 score (harmonic mean of precision, i.e., the fraction between true positives and predicted positives; and recall, i.e., the fraction between true positives and the target class cardinality). Both measures are typical when assessing the performance of classification algorithms. We have chosen to model only the *Single boom‐bust* due to the size of the dataset, which would prevent meaningful modeling for the least represented classes, such as *Multiple boom‐busts*, *Decline*, and *Rise*.

To quantify boom and bust synchronization within a patch and assess whether there was a patch signature (local environmental conditions across years affecting all local breeding species) in the occurrence of BBD, we rely on event coincidence analysis (Quiroga et al., 2002), a method that has seen a broad range of applications from neuroscience (Pereda et al., 2005), to climate (Stolbova et al., 2014), and sociology (Garcia et al., 2025). Let us consider two time series of events, $s_{x}=\left\{t_{x}^{1},t_{x}^{2},t_{x}^{3},\ldots ,t_{x}^{n}\right\}$ and $s_{y}=\left\{t_{y}^{1},t_{y}^{2},t_{y}^{3},\ldots ,t_{y}^{m}\right\}$, where $t_{k}^{i}$ represents the time stamp of the ith event for the time series k. Using the Hungarian algorithm, the events in the two series are paired to minimize the total time difference between them. Then, we fix a window of maximum overlap τ that represents the maximum time difference between two events to be considered coincident. This builds a boolean n×m matrix J with elements:

$$J_{\mathrm{ij}}^{\tau }=\left\{\begin{array}{l} 1\;\;\text{if event}\;i\;\text{in series}\;x\;\text{is coincident with event}\;j\\ \;\text{in series}\;y\\ 0\;\;\text{otherwise} \end{array}\right.$$

Then, the event coincidence index $Q_{\tau }$ is simply

$$Q_{\tau }=\frac{1}{\sqrt{m\;n}}\sum_{\mathrm{ij}}J_{\mathrm{ij}}^{\tau }.$$

This index ranges from 0 (no coincidence at all) to 1 (all events in the two series are coincident). Note that to have $Q_{\tau }=1$, the number of events in the two series must be equal (n=m).

In the time series under analysis, we deal with two classes of events. The first represents regime changes towards higher abundances, while the second represents regime changes towards lower abundances. These two classes of events have to be treated separately when assessing their coincidence. To obtain a single index for a pair of time series, we define the overall coincidence index as a weighted average of the two:

$${\bar{Q}}_{\tau }=\frac{\sqrt{m_{+}\;n_{+}}\;Q_{\tau }^{+}+\sqrt{m_{-}\;n_{-}}\;Q_{\tau }^{-}}{\sqrt{m_{+}\;n_{+}}+\sqrt{m_{-}\;n_{-}}}$$

where the weights consider the different numbers of events for the two types.

A final level of coincidence, $\left\langle {\bar{Q}}_{\tau }\right\rangle$, is assigned to the whole patch as the average of all ${\bar{Q}}_{\tau }$ values for all possible pairs of time series in the patch.

To assess the statistical significance of each value of $\left\langle {\bar{Q}}_{\tau }\right\rangle$, we employ surrogate analysis. For each pair of time series, we randomly shuffle the events of one of them and repeat the calculation of $\left\langle {\bar{Q}}_{\tau }\right\rangle$ 1000 times. From the randomized ${\bar{Q}}_{\tau }$ values, we extract empirical *p*‐values to assign to the measured $\left\langle {\bar{Q}}_{\tau }\right\rangle$.

Finally, to determine whether the bust phases and overall fluctuations in the time series result from breeding individuals redistributing across the patches available in the dataset, we computed the correlation coefficients between the yearly time series differentials of a single species in a given patch and those of the aggregated time series for all other patches. If a species emigrates from one patch and redistributes among the others, we would expect to observe anticorrelations in the differentials, as a decline in one patch should correspond to an increase in the rest, and vice versa. However, redistribution may also occur toward patches outside the study area, particularly farther south, given the high mobility of the species under consideration.

### Abundant and less abundant species

To study which species were able to persist after colonization, we focused on the dynamics of rare and less common species separately. We considered as unsuccessful invaders those cases that failed to establish a long‐lasting presence in the patches. We use the term “extinction” to refer to the local disappearance of a species from a patch, regardless of whether recolonization later occurred. As such, recurrent colonizations imply one or more preceding local extinctions. We set an arbitrary threshold of 1000 females over the study period within a given patch (i.e., species–patch combinations) above which a time series is considered an “abundant species” and below which is considered a “less abundant” species. While this cutoff is necessarily arbitrary, it provides a necessary distinction between small and large populations, whose dynamics are likely driven by different underlying demographic and ecological processes.

In particular, these rare species presented challenges for regime detection. Their time series often represented intermittent colonization episodes, typically lasting a single year. Analyzing these data through an algorithm of regime detection would either produce an unrealistic series of numerous regimes or condense the series into a single constant regime. Therefore, regime detection was applied to abundant species only. For less abundant species, we focused on invasion events, specifically their number and duration. We used the Kruskal–Wallis *H* test to assess whether distributions of invasion properties varied across categories defined by time series metadata.

## RESULTS

**FIGURE 3:**
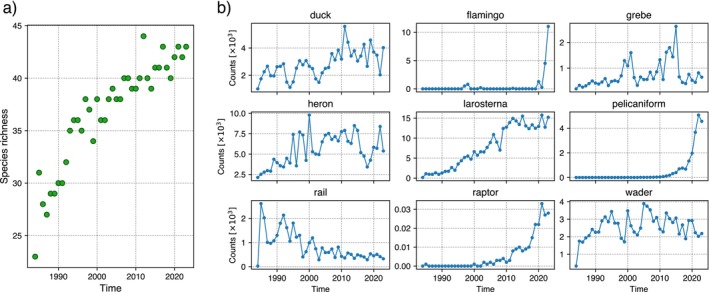
(a) Total number of species across the 53 patches under analysis as a function of time. (b) Abundances of time series for the waterbird ecological typologies aggregated across all the patches under analysis. Duck aggregates 10 different species, rail 2 species, flamingo 1 species, grebe 3 species, heron 8 species, larosterna 10 species, pelecaniformes 3 species, raptor 1 species, and wader 9 species. Most time series present nonstationary transient behaviors, generally out of equilibrium.

Despite the considerable time elapsed since the study patches were subjected to legal protection and recolonization began, the system still seems to be out of equilibrium for both species abundance in each patch and the total number of species. Species richness shows an exponential‐like increase since the early years (Figure 3a). Although the rate has slowed, it continues to rise, making its eventual stabilization hard to predict.

Most aggregated time series of the bird typologies exhibit clear signs of transient behavior (Figure 3b). The time series averages display long‐term trends and/or outlier fluctuations, clear signs of dynamics that are not yet at equilibrium. Some groups, such as raptors and flamingoes, have only recently begun colonizing the region. Others, like waders and ducks, have been present since the earliest years of monitoring but display large fluctuations. Herons and grebes show extreme bursts, sometimes tripling density before rapidly declining to previous levels.

### Abundant species

**FIGURE 4:**
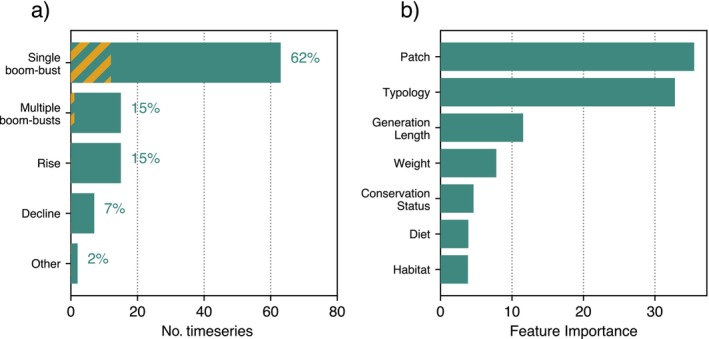
(a) Classification outcomes for the 102 time series corresponding to the most abundant species. Bars show the number and proportion of time series assigned to each population dynamic pattern. The hatched segment within the “boom–bust” bar highlights the subset identified as Canonical boom–bust dynamics. (b) Feature importance of the gradient boosting algorithm classifying *Single boom‐bust* time series based on time series metadata.

We began our analysis with the most abundant species, comprising 102 time series. We found that 62% of the time series followed a Single boom–bust pattern, 15% a Multiple boom–busts pattern, 15% a *Rise* pattern, 7% a *Decline* pattern, and 2% an Other pattern (Figure 4a). Among the time series associated with BBD, 13 (17%) were identified as canonical, of which one belongs to *Multiple boom‐busts* (Appendix S1: Table S2 and Figure S1). Over two thirds of the time series display non‐monotonous behaviors associated with transient dynamics. Logistic growth, which is a possibility included in the class *Rise*, affects only a minority of the species that successfully invaded the patches.

The χ^2^ tests revealed an association with diet ($\chi_{4}^{2}=9.6$, p=0.048), and its frequency anomaly analysis indicates that generalist species exhibit higher frequencies of *Rise* and *Single boom–bust* dynamics (Appendix S1: Tables S3 and S4). In comparison, specialist species show higher frequencies of *Decline* and *Multiple boom‐busts*. We found an association between the dynamics pattern and the bird typology ($\chi_{24}^{2}=43.3$, p=0.0092, $\chi_{28}^{2}=45.4$, p=0.020). In particular, grebes displayed a tendency to *Multiple boom‐busts*, waders showed low frequencies of *Rise* and a high occurrence of *Single boom‐bust*, whereas rails exhibited *Decline* frequencies significantly higher than expected (Appendix S1: Table S5). The χ^2^ test also reveals an association with conservation status. We treated conservation status as both a categorical and ordinal variable, performing χ^2^ and Kruskal–Wallis tests, respectively. Interestingly, only the χ^2^ test yielded a significant association; in particular, the *Rise* class is significantly more frequent in near‐threatened species than in least‐concern species. This is probably due to the fact that flamingos (a near‐threatened species) systematically display a *Rise* across all patches. Finally, the Kruskal–Wallis *H* test revealed a statistically significant relationship with generation time (H=14.9, p=0.005, n=4), with species classified as *Rise* having longer generation times, while no association emerged regarding body mass (H=6.38, p=0.17, n=4) (Appendix S1: Table S7). Detailed results of the statistical tests are reported in Appendix S1 (Tables S3–S10).

Regarding the use of multiple explanatory variables to classify the time series, the gradient‐boosting algorithm's performance on the test set yielded an accuracy of 71% and an F1 score of 77%, slightly above the random benchmark of 62%. This indicates weak generalization, likely due to the small dataset and limited feature predictive power. However, the model still performs better than random, suggesting that it captures some relevant information for classifying *Single boom‐bust* time series. The features and their relevance returned by the algorithm are shown in Figure 4b. As illustrated, the most informative driver appears to be the patch, followed by typology. While typology was already highlighted in the univariate analysis, the patch emerges as a relevant variable only in the multivariate analysis, implying that it contributes to the classification in a nonlinear manner, possibly through interactions with other drivers.

A significant association emerged for the patch for the occurrence of *Canonical boom–bust* dynamics ($\chi_{12}^{2}=24.6$, p=0.017). In particular, *Marjal de Almenara* and *Marjal dels Moros* displayed significantly higher occurrences than expected, while *Santa Pola* salt pans and *El Hondo* displayed significantly lower occurrences than expected (Appendix S1: Table S9). Given this patch‐specific association, we hypothesized a connection between *Canonical boom–bust* dynamics and climatic variability. Only sporadic and nonsystematic co‐occurrences between canonical *Single boom–bust* events and anomalous rainfall records were observed.

**FIGURE 5:**
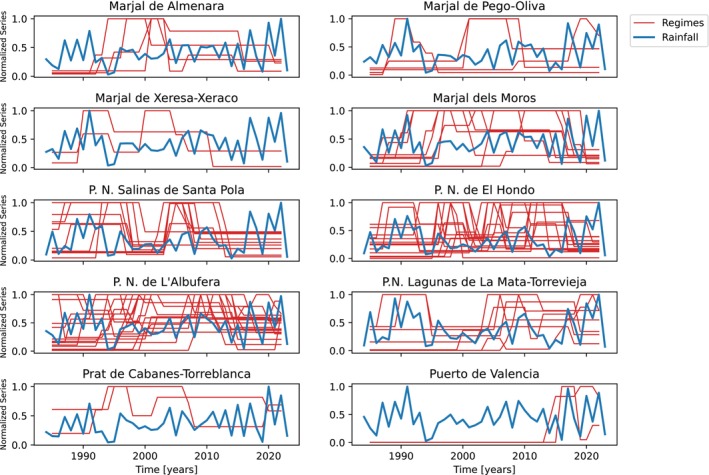
Abundance regimes (red thin lines) and rainfall (blue thick line) for each patch presenting at least two species with at least one boom–bust event. All time series are rescaled so that their maximum is 1.

In Figure 5, we show the boom‐bust regimes and the rainfall time series for 10 patches. Boom and bust regimes do not seem to coincide with rainfall anomalies. If rainfall had a significant impact on time series abundances, its effect would be even more evident on the aggregated counts per patch, as the independent noise in the time series would average out, increasing the signal‐to‐noise ratio. However, in Table S11 (see Appendix S1), no significant correlation can be detected between the two quantities, the highest being 0.1553 with a *p*‐value of 0.3385 in *Marjal de Pego‐Oliva*.

**FIGURE 6:**
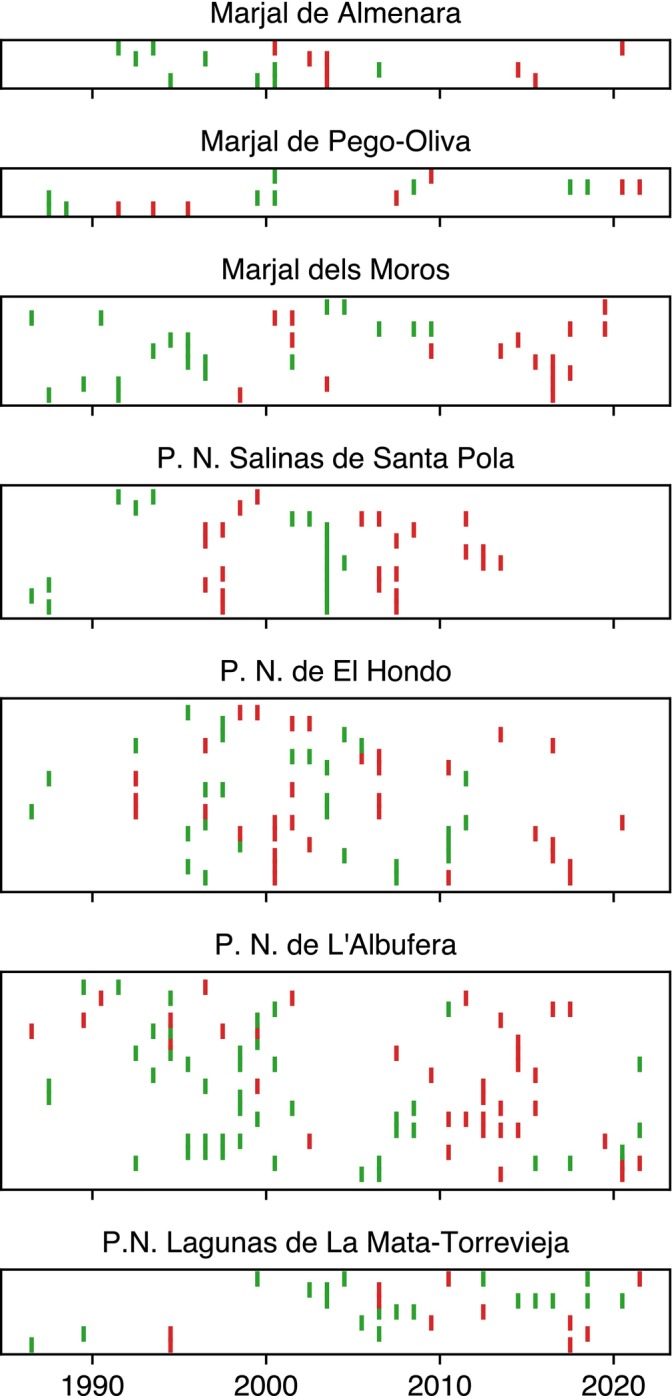
Regime shift occurrences for patches hosting more than two species displaying boom–bust‐related dynamics. For each patch, species are arranged along the vertical axis. A vertical green line represents a regime shift marked by an increase in the species' mean abundance, whereas a red vertical line means a regime shift toward lower abundances. The seven panels display different heights because they host different numbers of abundant species.

As a broader test for external drivers of BBD, we examined whether boom–bust events among species within a single patch occurred in phase (i.e., were synchronized). Figure 6 presents the detected regime shifts for all those patches having more than two species displaying BBD, either multiple or single. Only *P. N. Salines de Santa Pola*, *El Hondo*, *L'Albufera*, and to a lesser extent *Marjal dels Moros*, show significant synchronization, with clear alternation of booms and busts (Figure 6, Table 1). This suggests that, at least for these patches, the occurrence of BBD might be affected by external, nonclimatic drivers. For all other patches, no clear general co‐occurrence patterns can be spotted.

**TABLE 1:** Results of the coincidence analysis for τ=0 and τ=1.

Patch	τ=0		τ=1	
	$\left\langle {\bar{Q}}_{\tau }\right\rangle$	*p*‐value	$\left\langle {\bar{Q}}_{\tau }\right\rangle$	*p*‐value
Marjal de Almenara	0.15	0.049	0.27	0.061
Marjal de Pego‐Oliva	0.1	0.13	0.1	0.55
Marjal dels Moros	0.099	0.015	0.19	0.034
P. N. Salinas de Santa Pola	0.24	0.001	0.41	0.001
P. N. de El Hondo	0.1	0.001	0.23	0.001
P. N. de L'Albufera	0.082	0.020	0.22	0.001
P. N. Lagunas de La Mata‐Torrevieja	0.071	0.33	0.17	0.26

**FIGURE 7:**
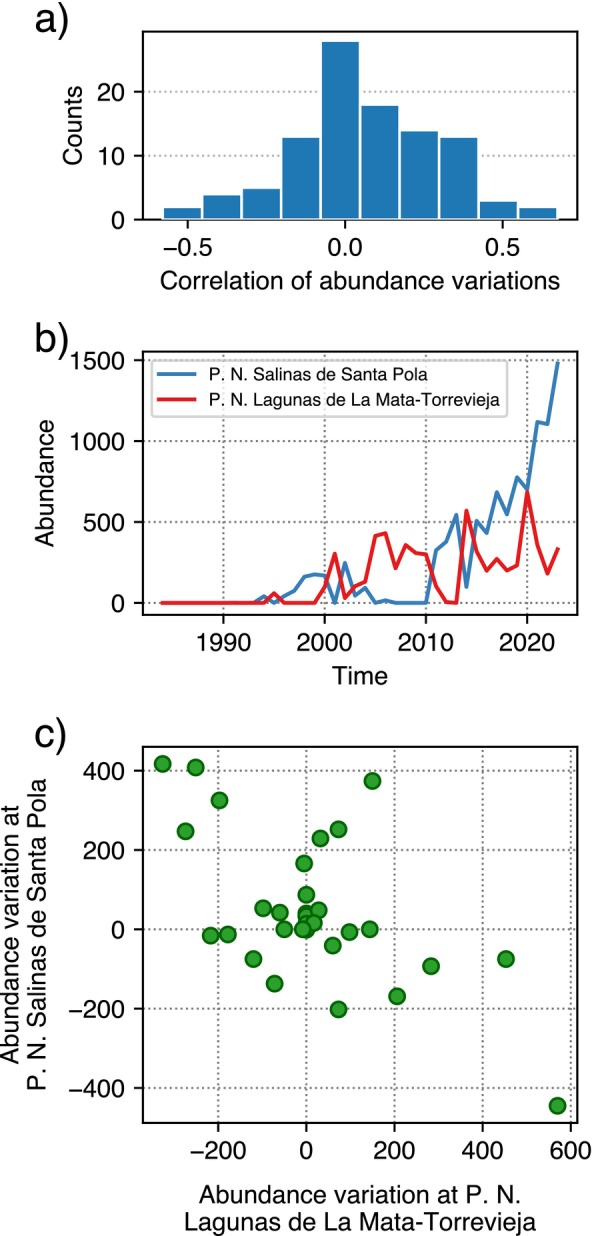
(a) Correlations between abundance variation in consecutive years for a single species in a single patch and the cumulative abundance variation of the same species in all the other patches. (b) Abundances of *Larus genei* in two patches showing alternate occupation. (c) Scatterplot of the abundance variation in consecutive years for the time series in panel (b). A strong anticorrelation was detected (r=−0.60, n=39, $p=6.0\times 10^{-5}$).

Positive correlations of the differentials for the 102 time series analyzed were prevalent, which aligns with the expectation that patches were recolonized simultaneously (Figure 7a). The only statistically significant anticorrelation occurred in *Larus genei*, a species exhibiting a negative correlation between two patches and the rest ($r_{1}=-0.58$, $p_{1}=1.1\times 10^{-4}$, $N_{1}=39$; and $r_{2}=-0.56$, $p_{2}=2.0\times 10^{-4}$, $N_{2}=39$) (Figure 7b). Specifically, this species seems to oscillate between *P. N. Salinas de Santa Pola* and *P. N. Lagunas de La Mata‐Torrevieja*, two patches located just 20 km apart (Figure 7c). However, this remains an exception, and overall, there is no strong evidence supporting a redistribution of the most abundant species across the patches.

### Less abundant species

**FIGURE 8:**
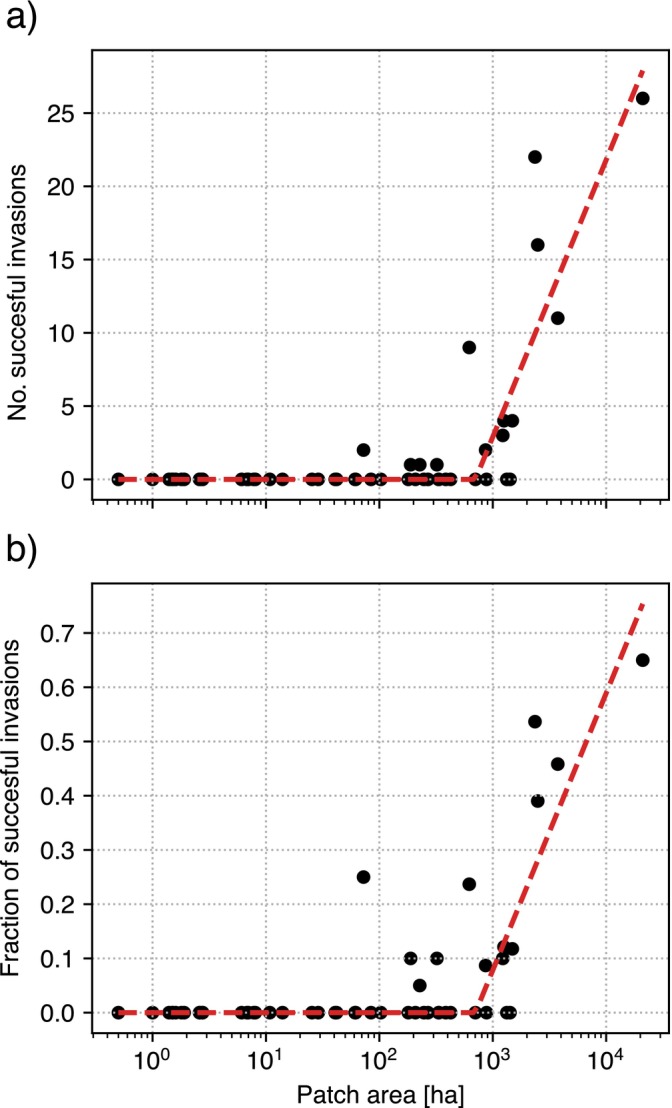
(a) Number of time series categorized as “successful invasions” as a function of the patch area. (b) Fraction of successful invasions over all time series as a function of the patch area. In both panels, the red dashed line represents a broken‐line fit of the model $y=\max\left\{0,\text{mlog}\left(x/b\right)\right\}$, where x is the patch area and b the breakpoint area. Values of m and b are obtained by minimizing the RMSE between model and data. For panel (a) we have b=703 ha, m=8.21, RMSE =2.59, n=52. For panel (b), instead, b=703 ha, m=0.22, RMSE =0.0753, n=52.

The number of time series having a cumulative count less than 1000 is 538, which is approximately five times higher than the number of time series of abundant species. Even when restricting the comparison to the 13 patches that also host abundant species, there are still 250 time series in this set, which is more than twice the number of abundant species in those same patches. The main driver impacting the chances that a time series is abundant or less abundant seems to be related to the patch area (Figure 8). Both the fraction and the absolute number of abundant species display a linear dependence on the logarithm of the patch size, with a lower cutoff at approximately 600—800 ha.

**FIGURE 9:**
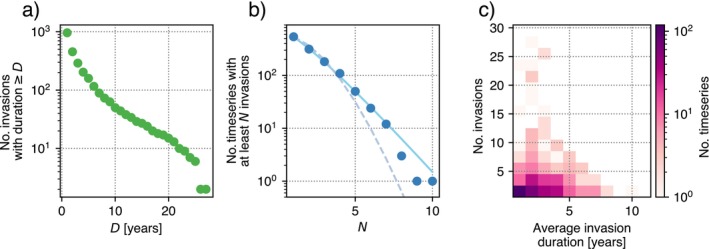
(a) Cumulative distribution of invasion lengths (in years = *D*) for less abundant species. (b) As blue dots, the cumulative counts of the number of invasions in a given time series; as a solid line, the fit with a negative binomial distribution ($\chi_{7}^{2}=6.10$, p=0.53); as a dashed line, the fit with a Poisson distribution ($\chi_{7}^{2}=75$, $p=1.4\times 10^{-13}$). (c) Relationship between the number of invasions and average invasion duration for each time series.

In Figure 9a,b, we report the cumulative distributions of invasion duration and number of invasions per species in a given patch. The distribution of invasion durations is affected by a cutoff given by the finite length of the time series under analysis, while the cutoff in the number of invasions is less severe. Invasion duration follows approximately an exponential distribution, with an average of 4.3 years per invasion. The number of invasions, instead, is compatible with a negative binomial distribution. The two main mechanisms that can act to generate a negative binomial distribution are either overdispersion, given by temporal clustering, or a random count process with a random occurrence rate. To rule out the first hypothesis, we checked for autocorrelation in the invasion times and found none (r=0.091, p=0.074, n=382). There was no significant correlation between the number of invasions attempted by a single species in a single patch and the average duration of those invasions (Figure 9c). The majority of species invaded the patches under analysis sporadically, with invasions lasting only a few years.

A statistically significant effect of bird typology was observed on the invasion durations (H=16.9, $p=3.2\times 10^{-2}$, n=8). In particular, herons exhibited longer‐than‐average invasions, while gulls and terns had shorter‐than‐average durations. Habitat also significantly influenced the length of the invasion (H=12.0, $p=5.2\times 10^{-4}$, n=1), with freshwater habitats associated with longer invasions compared to saltwater habitats. Bird typology was also associated with the number of invasions (H=16.4, $p=3.7\times 10^{-2}$, n=8). In particular, gulls, terns, and waders display, on average, more invasions, while raptors are characterized by fewer ones.

Finally, a statistically significant but weak positive Spearman's rank correlation was observed between invasion duration and body mass ($r_{s}=0.11$, $p=5.2\times 10^{-4}$, n=960). This indicates a monotonic, albeit weak, relationship between the two, with heavier species tending to display longer periods of unsuccessful invasions.

## DISCUSSION

Here, we explored how restoration‐driven recolonization triggered BBD in a native waterbird community across 53 Mediterranean wetlands from 1984 to 2023. Although most studies on BBD focus on alien species (Strayer et al., 2017), our work highlights that native species (either expanding their distribution range or recolonizing patches within their historical range) can also exhibit BBD—a phenomenon that remains poorly studied (Lubina & Levin, 1988). Indeed, our results suggest that BBD can emerge solely from intrinsic ecological processes, without invoking exotic traits or maladaptation to the new habitat. This implies that some of the BBD observed in alien species invasions may reflect general ecological dynamics (e.g., demographic interactions) rather than the effect of alien functional traits, calling for a reinterpretation of BBD patterns often attributed to ecological novelty or temporal lack of biotic resistance (Johnson et al., 2023; Strayer, 2012).

Even though the system of patches under analysis has been protected for more than 40 years, encompassing three to eight generation times of the breeding species, recolonization is still underway, and species richness has nearly doubled without sign of saturation, instead exhibiting a long transient far from asymptotic dynamics. At the single species level, population dynamics were also far from stationarity and logistic shape, displaying nonlinear behaviors aside from short‐term stochasticity, while monotonic shapes characterized only a minority of the time series (Martínez‐Abraín et al., 2016). Among successful recolonizations, most show BBD (77% of the cases), either single (the prevalent dynamic with 62% of occurrences) or multiple booms. However, only 17% of the BBD cases belonged to the paradigmatic example often found in the literature of a steep rise followed by a steep bust after a relatively short time (Strayer, 2012; Strayer et al., 2017). We cannot rule out that this difference may be partially explained by the fact that our study species were native and not alien, but more research may be addressed in this direction.

Such nonlinearities commonly found in our study may result from several interacting ecological processes. For instance, immigration from outside the system may be pulsed and hampered by Allee effects (Johnson et al., 2006; Reigada et al., 2015; Schreiber & Lloyd‐Smith, 2009). Furthermore, most of the study species are adapted to breed in Mediterranean wetlands with high environmental stochasticity across years, which results in a nomadic reproductive behavior characterized by group dispersal (Francesiaz et al., 2020; Oro, 2002; Oro et al., 2004, 2009, 2023; Pacheco‐Guardiola et al., 2026). Invasion dynamics may also involve ecological fitting, even among native species, because invaders can establish in habitats where their existing functional traits already allow them to survive and reproduce, even without a history of local coevolution. Nevertheless, little is known about whether colonization into newly suitable habitats requires active resource tracking, relies mainly on preadapted trait–environment matching, or leads to novel biotic interactions (Agosta & Klemens, 2008). In our system, patch suitability improved by enhancing environmental conditions (e.g., water quality, harvesting). In strictly protected patches, this improvement was more abrupt, whereas in the remaining patches, conditions tended to improve more gradually following the broader implementation of environmental regulations. Thus, the observed BBD is more likely driven by demographic and environmental processes related to the formation of associations (e.g., interference competition and exclusion) during succession and community reassembly (Oro et al., 2009; Pagel et al., 2014).

### Drivers in boom–bust dynamics

While a variety of ecological and demographic mechanisms have been hypothesized to explain the emergence of BBD (Arthington & Balcombe, 2011; Duncan et al., 2020; Iles et al., 2016; Keane & Crawley, 2002; Oro et al., 2023; Strayer, 2012), we focus here on the environmental and species‐level attributes that determine where and when BBD occurred. The main driver of the invasion process and BBD was likely the effective protection of patches, when the Spanish habitat protection law aligned with international directives soon after the study onset. This change represented a step change, a type of single rare event that can trigger profound ecological responses by abruptly altering ecosystem features (Oro et al., 2024; Ryo et al., 2019), and more precisely, habitat suitability (Duncan et al., 2020; Martínez‐Abraín et al., 2016; Martínez‐Abraín et al., 2019; Pagel et al., 2014). Patches freed from major anthropogenic pressures (e.g., uncontrolled hunting and eutrophication) and invasive predators should, in general, facilitate succession, enhancing colonization and population growth as in classic post‐disturbance dynamics (Margalef, 1963; Poorter et al., 2023; Sousa, 1979). Such anthropogenic, deterministic changes can act as a bifurcation point and push ecosystems across thresholds into new dynamic regimes (Oro & Martínez‐Abraín, 2023; Suding & Hobbs, 2009). Some stressors, such as agricultural chemicals, still affect patches (especially shallow ones) under eutrophication, likely prolonging the transient to stationarity (Hastings et al., 2018; Schreiber & Lloyd‐Smith, 2009; Vera‐Herrera et al., 2021). Regulated and sporadic illegal hunting in the study area remains ongoing (Crespo et al., 2020; Martínez‐Abraín et al., 2007, 2009; Pérez‐García et al., 2024; Tavecchia et al., 2009), and notably, the species most exposed to this perturbation (most ducks and coots) are the ones showing the greatest population declines at the patch level (see Table S5). This pattern suggests that, along with other processes (e.g., metapopulation dynamics), human harvesting of invasive species may obscure signals of BBD, transient phenomena, and other forms of nonlinear population behavior (Martínez‐Abraín et al., 2016).

Consistent with both conceptual frameworks and invasion case studies, the emergence of BBD in our study was not uniform across all species and patches (Duncan et al., 2020; Southwood et al., 1974; Strayer et al., 2017). For instance, generalist species were more likely to show *Rise* and single BBD, while specialist species (i.e., with narrow trophic requirements) showed more declining trends and multiple BBD (Martínez‐Abraín et al., 2016), in agreement with the suggestion that trophic plasticity may stabilize population dynamics (Moor et al., 2021; Richmond et al., 2005). Besides, patches exhibiting more variability in hydrological regimes produced more canonical BBD, whereas more stable or heavily managed habitats (e.g., harbors, salt pans) were less prone to these dynamics. Strikingly, rainfall was not directly related to boom–bust events, likely because anthropogenic ecosystem management may obscure the climatic signal (Vera‐Herrera et al., 2021; Zhang et al., 2021). In particular, some patches in harbors and other human‐made infrastructures do not experience significant water‐level fluctuations, thereby providing a relatively stable habitat, even during droughts. Thus, higher stochasticity in environmental conditions may be a significant, commonly unnoticed driver of BBD (Arthington & Balcombe, 2011). The variation across species and patches not only affects the BBD occurrence but also the size and duration of the peak phase and the severity of the subsequent bust. This heterogeneity suggests diverse drivers and mechanisms affecting one species–patch combination may differ from another (Oro, 2013). A further possibility is that one single mechanism is responsible for the nonlinearity, but its strength and duration would be modulated across species and patches, causing the different shapes observed. In line with this lack of a canonical shape, patch features and species attributes, such as ecological typology, life history, and trophic plasticity, emerged as interacting drivers in the occurrence of BBD. It has been suggested that patches with limited resources (e.g., food, space) may enhance the appearance of BBD in invasive species (Turchin, 2003), but some studies emphasize that it is the interaction of the environmental features of the patch with the traits of the species that shapes such dynamics (Dijoux et al., 2024; Johnson et al., 2023; Moor et al., 2021). For instance, species with slower evolutionary life histories (i.e., with longer generation times) and colonizing more stable patches showed less BBD and more *Rise* dynamics, likely because their populations buffer environmental stochasticity (Duncan et al., 2020; Papacostas et al., 2017; Roff, 1992; Stearns, 1992).

### Most invasions conclude as unsuccessful events

Most general studies on invasion ecology focus on successful invasions, whereas failed events, their occurrence and causes are missed or not dealt with extensively (Lockwood et al., 2013; Simberloff & Rejmánek, 2011). Some other studies highlight the importance of comparing the characteristics of successful invasions with those of failures at the same stage to reach a reliable unified framework for biological invasions (Blackburn et al., 2011; Lockwood et al., 2005). Although some authors assume that most invasions are unsuccessful (Blackburn et al., 2011, Lockwood et al., 2005), their occurrence has been little quantified (Heersink et al., 2020; Miller et al., 2007; Williams et al., 2021; Williamson, 1996). Yet, many invasions may go undetected, and this can be a source of biases which challenge detailed studies on that occurrence and its drivers (Papacostas et al., 2017; Zenni & Nuñez, 2013). In our study on native species (which should be more adapted to successfully invade a patch), unsuccessful invasion has been rather the rule (~80% of cases), likely reflecting that colonizing propagule sizes were commonly small (especially in small patches), which may generate Allee effects (e.g., social cues such as social copying and both conspecific and heterospecific attraction) (Camacho‐Cervantes et al., 2023; Oro, 2020; Simberloff, 2009). As a result, several patches remained substantially unexploited by our study species. This suggests that the invasion process at large spatial scales, encompassing metapopulations and spatially structured populations, may shape both population and range dynamics (Andrade‐Restrepo et al., 2019; Grayson & Johnson, 2018).

Interestingly, we found that the number of unsuccessful invasions per time series follows roughly a negative binomial distribution, while their duration is roughly exponential. Overall, short, sporadic invasions involving small propagule size are the most common scenario. Moreover, exponentially distributed invasion durations might also indicate a substantial lack of temporal correlation between the events. Taken together, these results suggest that unsuccessful invasions likely act as a background noisy process (Turchin, 2003). Experimental and theoretical evidence have shown that randomness and low propagule pressure drive frequent invasion failures. In insects, for instance, Allee effects for mating reduce establishment probability (Williams et al., 2021), while in microbial systems, stochastic failures occur even when invaders closely resemble residents in functional traits (Kinnunen et al., 2018). Small patches, with an inherent short availability of resources (e.g., food, shelter), tend to experience invasions with smaller propagule size and consequently, more noisy dynamics. Indeed, patches smaller than 200 ha do not show any successful invasion at all. Nonetheless, a detectable phylogenetic signal implies that evolutionary lineage (e.g., bird typology, life history) modulates this stochasticity. Such modulation can act in multiple ways, such as reducing demographic fluctuations, varying levels of social copying, or enhancing resilience to environmental variability (Capdevila et al., 2022).

## CONCLUSIONS

Our findings highlight the complexity of invasion population dynamics, even for native species that recolonize restored patches. Successful invasions constitute relatively improbable events emerging from the noisy background of attempted colonizations. Moreover, they are generally characterized by long, nonlinear transients on a scale spanning several generation times, even for highly mobile species, such as birds, that can adjust quickly through prospecting dispersal to environmental changes. In particular, BBD emerges as a rather ubiquitous dynamic, even though heterogeneous in strength and duration, challenging the prevalent idea that it should be a phenomenon related to alien species invasion. The interplay between phylogenetic and geographic signals highlights how the drivers behind such transients can be diverse and might require a case‐by‐case study to be fully characterized.

## AUTHOR CONTRIBUTIONS

Daniel Oro devised the project. Daniel Oro collected some of the data. Giulio Tirabassi performed the analysis. Daniel Oro and Giulio Tirabassi wrote the manuscript.

## CONFLICT OF INTEREST STATEMENT

The authors declare no conflicts of interest.

## Supporting information


Appendix S1.

## Data Availability

Data are available in the Biodiversity Data Bank of the Valencian Community at https://bdb.gva.es/es/censos-d-aus-aquatiques. Metadata are available in DIGITAL.CSIC at http://hdl.handle.net/10261/418812.
