# Supplementary material for: Stochastic colonization, transient and boom–bust dynamics shape invasions by native taxa
Source: Ecology. 2026 May 6;107:e70398. doi: 10.1002/ecy.70398 (PMC13150415; doi:10.1002/ecy.70398)
Supplement: Supplementary file 1 — Appendix S1. [file ECY-107-e70398-s001.pdf]

Appendix S1

Stochastic colonization, transient and boom–bust dynamics shape invasions by native taxa

Daniel Oro, Giulio Tirabassi

Ecology

Section S1: Species metadata

The following table (Table S1) compiles the metadata for each one of the 47 species in the dataset.

Table S1: Waterbird species under analysis.

Species	Diet	Habitat	Typology	Status	Generation time [years]	Weight [g]
Anas clypeata	SP	FW	duck	LC	NaN	735
Anas platyrhynchos	GE	FW	duck	LC	4.781013	1150
Anas querquedula	SP	FW	duck	CR	NaN	450
Anas strepera	SP	FW	duck	LC	NaN	900
Ardea cinerea	GE	FW	heron	LC	8.879565	1545
Ardea purpurea	GE	FW	heron	NT	6.351622	1000
Ardeola ralloides	GE	FW	heron	VU	4.137541	300
Aythya ferina	SP	FW	duck	NT	4.227950	1000
Aythya nyroca	SP	FW	duck	CR	3.514308	700
Bubulcus ibis	GE	FW	heron	LC	6.069602	391
Burhinus oedicnemus	GE	FW	wader	NT	7.434048	470
Casmerodius albus	GE	FW	heron	NT	NaN	950
Charadrius alexandrinus	SP	SA	wader	EN	4.057965	37
Charadrius dubius	GE	FW	wader	LC	3.355648	35
Chlidonias hybrida	GE	FW	larosterna	VU	4.732208	70
Circus aeruginosus	GE	FW	raptor	LC	NaN	645
Egretta garzetta	GE	FW	heron	LC	4.243233	350
Fulica atra	SP	FW	rail	LC	4.196714	750
Fulica cristata	SP	FW	rail	CR	3.917559	1000
Gelochelidon nilotica	GE	SA	larosterna	VU	9.747824	200
Glareola pratincola	SP	SA	wader	VU	3.854532	150
Himantopus himantopus	GE	FW	wader	LC	5.599142	200
Ixobrychus minutus	GE	FW	heron	LC	3.333238	140
Larus audouinii	GE	SA	larosterna	VU	10.650585	550
Larus genei	SP	SA	larosterna	NT	9.640456	330
Larus melanocephalus	GE	FW	larosterna	NT	7.494382	300
Larus michahellis	GE	FW	larosterna	NT	11.605591	900
Larus ridibundus	GE	FW	larosterna	LC	9.848458	350
Marmaronetta angustirostris	SP	FW	duck	CR	2.941435	600
Netta rufina	SP	FW	duck	LC	2.497672	1200
Nycticorax nycticorax	GE	FW	heron	NT	5.994405	800
Oxyura leucocephala	SP	SA	duck	EN	4.605350	600
Phalacrocorax carbo	GE	FW	pelicaniform	LC	8.710598	2300
Phoenicopterus roseus	SP	SA	flamingo	NT	15.603474	3000
Platalea leucorodia	SP	FW	pelicaniform	VU	9.340385	1800
Plegadis falcinellus	GE	FW	pelicaniform	NT	7.246297	600
Podiceps cristatus	SP	FW	grebe	LC	5.448573	1250
Podiceps nigricollis	SP	SA	grebe	LC	5.027990	400
Porphyrio porphyrio	GE	FW	wader	NT	4.738940	1200
Recurvirostra avosetta	SP	SA	wader	LC	7.005610	300
Sterna albifrons	GE	SA	larosterna	NT	NaN	45
Sterna hirundo	GE	SA	larosterna	NT	8.397242	130
Sterna sandvicensis	GE	SA	larosterna	VU	NaN	210
Tachybaptus ruficollis	SP	FW	grebe	LC	4.650740	250
Tadorna tadorna	SP	SA	duck	LC	8.132459	1200
Tringa totanus	SP	SA	wader	LC	5.026408	150
Vanellus vanellus	GE	FW	wader	LC	6.185876	230

Section S2: Boom–bust events

The following table (Table S2) compiles statistics of the boom–bust events detected in the dataset. Namely:

- Boom duration: the number of years between the end of the baseline period and the beginning of the highest regime.
- Boom peak duration: the duration in years of the highest regime.
- Bust duration: the number of years between the last year of the highest regime and the first year of the lowest regime after the peak.
- Peak: average count of the highest regime.
- Boom baseline: average count of the lowest regime before the peak
- Bust level: average count of the lowest regime after the peak
- Boom intensity: absolute difference between peak and baseline regimes.
- Boom relative intensity: difference between peak and baseline regimes relative to the peak.
- Bust intensity: absolute difference between peak and bust regimes.
- Bust relative intensity: difference between peak and bust regimes relative to the peak.
- Rows marked with a "C" denote a canonical boom–bust event.

These metrics are visually displayed in Fig. S1

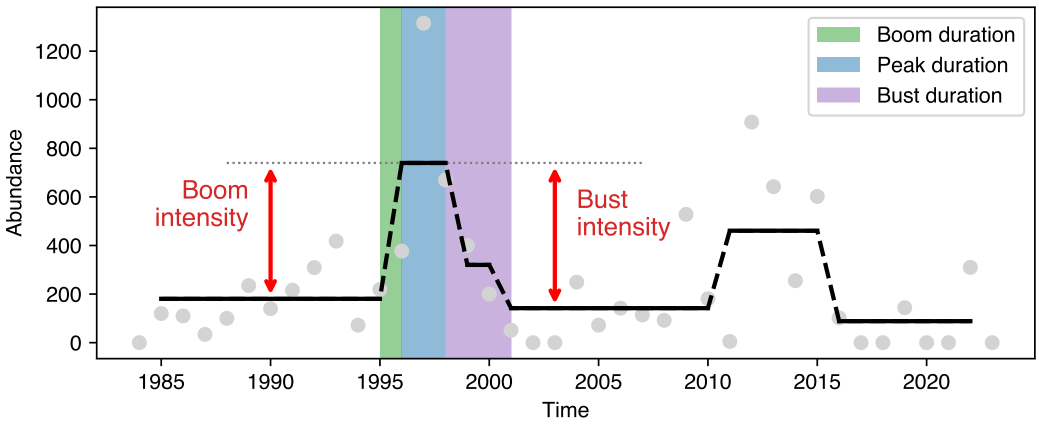

Figure S1: Key boom and bust metrics for a sample time series (*Chlidonias hybrida* in *P. N. de El Hondo*) displaying two events. Grey dots: females counts. Black line: detected regimes. The regimes present two local maxima; therefore, two boom and bust events are identified. Metrics are visually displayed only for the first event.

Table S2: Statistics of the boom–bust events detected in the dataset for each species and patch.

Species	Patch	Boom duration	Boom peak duration	Burst duration	Peak	Boom baseline	Bust level	Boom intensity	Boom rel. intensity	Bust intensity	Bust rel. intensity	
Himantopus himantopus	*Delta del Millars*	2	3	9	313.78	44.24	3.81	269.54	0.86	309.97	0.99	
Bubulcus ibis	*Hondo de Amorós*	6	2	2	320.00	0.83	8.33	319.17	1.00	311.67	0.97	C
Anas platyrhynchos	*Marjal de Almenara*	7	3	13	121.89	7.37	31.86	114.52	0.94	90.03	0.74	C
Chlidonias hybrida	*Marjal de Almenara*	1	3	1	163.78	15.42	14.56	148.36	0.91	149.22	0.91	
Chlidonias hybrida	*Marjal de Almenara*	1	8	1	60.21	14.56	14.42	45.65	0.76	45.79	0.76	
Fulica atra	*Marjal de Almenara*	5	6	2	83.33	3.33	24.07	80.00	0.96	59.26	0.71	C
Himantopus himantopus	*Marjal de Almenara*	3	7	21	143.43	7.86	33.17	135.57	0.95	110.26	0.77	C
Anas platyrhynchos	*Marjal de Pego-Oliva*	11	2	2	330.50	31.53	232.00	298.97	0.90	98.50	0.30	
Chlidonias hybrida	*Marjal de Pego-Oliva*	1	9	1	118.44	14.96	16.18	103.49	0.87	102.26	0.86	
Fulica atra	*Marjal de Pego-Oliva*	2	3	5	213.33	2.22	9.89	211.11	0.99	203.44	0.95	C
Himantopus himantopus	*Marjal de Pego-Oliva*	14	7	1	217.05	10.11	101.49	206.94	0.95	115.56	0.53	
Chlidonias hybrida	*Marjal de Xeresa-Xeraco*	1	5	17	80.13	6.67	1.64	73.47	0.92	78.49	0.98	
Fulica atra	*Marjal de Xeresa-Xeraco*	1	5	1	58.00	26.33	26.60	31.67	0.55	31.40	0.54	
Fulica atra	*Marjal de Xeresa-Xeraco*	1	5	3	97.60	26.60	28.50	71.00	0.73	69.10	0.71	
Anas platyrhynchos	*Marjal dels Moros*	5	7	19	126.71	9.33	25.33	117.38	0.93	101.38	0.80	C
Chlidonias hybrida	*Marjal dels Moros*	3	14	5	124.83	8.70	11.00	116.13	0.93	113.83	0.91	C
Fulica atra	*Marjal dels Moros*	3	12	14	95.56	15.33	20.50	80.22	0.84	75.06	0.79	
Glareola pratincola	*Marjal dels Moros*	1	20	2	53.08	8.47	11.40	44.61	0.84	41.68	0.79	
Himantopus himantopus	*Marjal dels Moros*	5	10	2	129.83	20.50	27.51	109.33	0.84	102.33	0.79	
Netta rufina	*Marjal dels Moros*	2	6	14	78.56	17.53	13.67	61.02	0.78	64.89	0.83	
Sterna albifrons	*Marjal dels Moros*	2	15	1	90.62	1.51	5.89	89.11	0.98	84.73	0.94	C
Sterna hirundo	*Marjal dels Moros*	4	8	3	208.79	4.65	31.78	204.14	0.98	177.01	0.85	C
Tachybaptus ruficollis	*Marjal dels Moros*	7	14	2	59.36	10.64	18.56	48.72	0.82	40.80	0.69	
Anas platyrhynchos	*P. N. Salinas de Santa Pola*	1	5	1	65.27	8.67	24.26	56.60	0.87	41.00	0.63	
Bubulcus ibis	*P. N. Salinas de Santa Pola*	2	8	2	1575.42	199.53	133.89	1375.89	0.87	1441.53	0.92	
Charadrius alexandrinus	*P. N. Salinas de Santa Pola*	1	10	1	268.83	179.33	47.72	89.50	0.33	221.11	0.82	
Charadrius alexandrinus	*P. N. Salinas de Santa Pola*	1	4	1	170.58	47.72	53.16	122.86	0.72	117.43	0.69	
Chlidonias hybrida	*P. N. Salinas de Santa Pola*	1	11	1	121.73	65.00	32.39	56.73	0.47	89.34	0.73	
Chlidonias hybrida	*P. N. Salinas de Santa Pola*	1	4	1	120.42	32.39	37.44	88.03	0.73	82.97	0.69	

Continued on next page

Species	Patch	Boom duration	Boom peak duration	Bust duration	Peak	Boom baseline	Bust level	Boom intensity	Boom rel. intensity	Bust intensity	Bust rel. intensity
Egretta garzetta	*P. N. Salinas de Santa Pola*	1	6	1	133.83	5.00	34.96	128.83	0.96	98.88	0.74
Himantopus himantopus	*P. N. Salinas de Santa Pola*	1	4	1	178.83	57.29	82.07	121.55	0.68	96.77	0.54
Larus michahellis	*P. N. Salinas de Santa Pola*	2	3	7	255.11	33.94	50.36	221.17	0.87	204.75	0.80
Larus ridibundus	*P. N. Salinas de Santa Pola*	3	6	1	310.61	48.05	153.12	262.56	0.85	157.50	0.51
Nycticorax nycticorax	*P. N. Salinas de Santa Pola*	1	8	2	103.50	15.18	5.77	88.32	0.85	97.73	0.94
Sterna albifrons	*P. N. Salinas de Santa Pola*	1	3	2	347.33	83.67	165.22	263.67	0.76	182.11	0.52
Tachybaptus ruficollis	*P. N. Salinas de Santa Pola*	1	9	1	35.04	11.56	5.33	23.48	0.67	29.70	0.85
Tachybaptus ruficollis	*P. N. Salinas de Santa Pola*	1	3	1	56.44	5.33	27.67	51.11	0.91	28.78	0.51
Anas platyrhynchos	*P. N. de El Hondo*	1	4	1	125.75	51.47	64.57	74.28	0.59	61.18	0.49
Anas platyrhynchos	*P. N. de El Hondo*	1	3	8	197.33	64.57	42.13	132.76	0.67	155.20	0.79
Aythya ferina	*P. N. de El Hondo*	1	5	1	332.73	85.79	126.38	246.95	0.74	206.35	0.62
Aythya ferina	*P. N. de El Hondo*	1	10	1	275.47	126.38	88.73	149.09	0.54	186.73	0.68
Charadrius alexandrinus	*P. N. de El Hondo*	1	6	5	157.78	58.33	34.05	99.44	0.63	123.73	0.78
Charadrius alexandrinus	*P. N. de El Hondo*	1	3	1	96.67	34.05	26.46	62.62	0.65	70.21	0.73
Chlidonias hybrida	*P. N. de El Hondo*	1	3	3	739.78	180.52	142.00	559.26	0.76	597.78	0.81
Chlidonias hybrida	*P. N. de El Hondo*	1	5	1	460.80	142.00	88.48	318.80	0.69	372.32	0.81
Egretta garzetta	*P. N. de El Hondo*	1	5	1	200.80	37.11	27.54	163.69	0.82	173.26	0.86
Glareola pratincola	*P. N. de El Hondo*	1	3	5	76.22	26.93	13.47	49.29	0.65	62.75	0.82
Himantopus himantopus	*P. N. de El Hondo*	1	4	1	230.92	97.33	97.59	133.58	0.58	133.32	0.58
Himantopus himantopus	*P. N. de El Hondo*	1	11	1	221.48	97.59	57.94	123.89	0.56	163.54	0.74
Ixobrychus minutus	*P. N. de El Hondo*	7	6	1	84.33	11.67	57.28	72.67	0.86	27.06	0.32
Larus ridibundus	*P. N. de El Hondo*	8	9	1	786.81	30.26	117.89	756.56	0.96	668.93	0.85
Oxyura leucocephala	*P. N. de El Hondo*	2	4	1	108.75	3.17	42.16	105.58	0.97	66.59	0.61
Phoenicopterus roseus	*P. N. de El Hondo*	1	3	2	342.78	0.00	3.91	342.78	1.00	338.86	0.99
Podiceps cristatus	*P. N. de El Hondo*	1	4	2	83.42	20.61	18.63	62.81	0.75	64.78	0.78
Podiceps cristatus	*P. N. de El Hondo*	1	9	1	54.41	18.63	24.67	35.77	0.66	29.74	0.55
Podiceps nigricollis	*P. N. de El Hondo*	1	4	1	388.33	21.60	184.29	366.74	0.94	204.04	0.53
Podiceps nigricollis	*P. N. de El Hondo*	1	6	1	820.83	184.29	113.28	636.54	0.78	707.56	0.86
Recurvirostra avosetta	*P. N. de El Hondo*	2	3	2	247.00	24.76	96.85	222.24	0.90	150.15	0.61
Sterna albifrons	*P. N. de El Hondo*	1	3	1	91.00	12.79	15.83	78.21	0.86	75.17	0.83
Tachybaptus ruficollis	*P. N. de El Hondo*	1	4	2	352.50	102.90	112.37	249.60	0.71	240.13	0.68
Ardea cinerea	*P. N. de L'Albufera*	12	14	1	564.33	87.33	330.20	477.00	0.85	234.13	0.41

Continued on next page

Species	Patch	Boom duration	Boom peak duration	Bust duration	Peak	Boom baseline	Bust level	Boom intensity	Boom rel. intensity	Bust intensity	Bust rel. intensity	
Ardeola ralloides	*P. N. de L'Albufera*	11	6	2	371.50	79.50	120.80	292.00	0.79	250.70	0.67	
Bubulcus ibis	*P. N. de L'Albufera*	1	16	7	2592.08	1273.30	954.90	1318.79	0.51	1637.18	0.63	
Egretta garzetta	*P. N. de L'Albufera*	10	3	14	1749.44	735.67	993.13	1013.78	0.58	756.31	0.43	
Glareola pratincola	*P. N. de L'Albufera*	2	7	8	85.95	12.95	26.33	73.00	0.85	59.62	0.69	
Himantopus himantopus	*P. N. de L'Albufera*	26	3	2	960.44	166.54	639.33	793.90	0.83	321.11	0.33	
Ixobrychus minutus	*P. N. de L'Albufera*	1	14	1	88.79	39.60	59.33	49.19	0.55	29.45	0.33	
Larus audouinii	*P. N. de L'Albufera*	2	4	3	539.42	7.84	13.81	531.58	0.99	525.61	0.97	C
Larus genei	*P. N. de L'Albufera*	9	3	3	139.89	2.47	16.13	137.42	0.98	123.76	0.88	C
Larus ridibundus	*P. N. de L'Albufera*	11	5	3	1121.47	34.64	713.24	1086.82	0.97	408.23	0.36	
Netta rufina	*P. N. de L'Albufera*	2	3	3	73.44	24.19	33.43	49.25	0.67	40.01	0.54	
Podiceps cristatus	*P. N. de L'Albufera*	1	7	11	53.38	37.92	22.00	15.46	0.29	31.38	0.59	
Porphyrio porphyrio	*P. N. de L'Albufera*	3	13	1	109.85	1.42	62.30	108.42	0.99	47.55	0.43	
Recurvirostra avosetta	*P. N. de L'Albufera*	4	4	18	89.83	2.94	48.00	86.89	0.97	41.83	0.47	
Sterna albifrons	*P. N. de L'Albufera*	3	5	1	158.53	17.00	79.14	141.53	0.89	79.39	0.50	
Sterna hirundo	*P. N. de L'Albufera*	7	9	8	2123.22	182.08	443.50	1941.14	0.91	1679.72	0.79	
Sterna sandvicensis	*P. N. de L'Albufera*	6	14	1	1641.36	28.45	690.38	1612.90	0.98	950.98	0.58	
Tachybaptus ruficollis	*P. N. de L'Albufera*	1	15	1	57.09	29.47	31.67	27.62	0.48	25.42	0.45	
Charadrius alexandrinus	*P.N. Lagunas de La Mata-Torrevieja*	1	8	24	178.21	76.67	22.20	101.54	0.57	156.01	0.88	
Gelochelidon nilotica	*P.N. Lagunas de La Mata-Torrevieja*	1	3	1	41.67	0.93	5.12	40.74	0.98	36.54	0.88	
Himantopus himantopus	*P.N. Lagunas de La Mata-Torrevieja*	1	5	1	132.87	56.93	35.47	75.93	0.57	97.39	0.73	
Himantopus himantopus	*P.N. Lagunas de La Mata-Torrevieja*	1	12	1	115.78	35.47	45.58	80.31	0.69	70.19	0.61	
Larus audouinii	*P.N. Lagunas de La Mata-Torrevieja*	3	4	1	2924.83	18.36	2094.33	2906.47	0.99	830.50	0.28	
Larus genei	*P.N. Lagunas de La Mata-Torrevieja*	6	6	1	310.83	6.18	86.00	304.66	0.98	224.83	0.72	C
Larus genei	*P.N. Lagunas de La Mata-Torrevieja*	7	3	1	402.00	86.00	290.33	316.00	0.79	111.67	0.28	
Recurvirostra avosetta	*P.N. Lagunas de La Mata-Torrevieja*	1	4	9	122.17	45.98	34.80	76.18	0.62	87.37	0.72	
Sterna hirundo	*P.N. Lagunas de La Mata-Torrevieja*	2	3	1	349.22	54.41	191.88	294.81	0.84	157.35	0.45	
Glareola pratincola	*Prat de Cabanes-Torreblanca*	1	12	2	64.11	39.10	20.03	25.01	0.39	44.08	0.69	
Tachybaptus ruficollis	*Prat de Cabanes-Torreblanca*	3	4	1	103.17	20.57	51.83	82.60	0.80	51.33	0.50	

Continued on next page

Species	Patch	Boom duration	Boom peak duration	Bust duration	Peak	Boom baseline	Bust level	Boom intensity	Boom rel. intensity	Bust intensity	Bust rel. intensity
Tachybaptus ruficollis	*Prat de Cabanes-Torreblanca*	1	18	1	84.33	51.83	60.22	32.50	0.39	24.11	0.29
Larus audouinii	*Puerto de Castellón*	4	5	3	3615.27	3.88	2499.50	3611.38	1.00	1115.77	0.31
Larus audouinii	*Puerto de Valencia*	6	3	1	858.67	2.75	702.67	855.92	1.00	156.00	0.18
Sterna sandvicensis	*Puerto de Valencia*	1	3	1	426.11	1.89	21.67	424.22	1.00	404.44	0.95

Section S3: Statistical tests results

Table S3: Results of the chi2 tests for association between regimes and different patch- and species-related variables. Asterisks mark results below the threshold of 5% significance.

variable	p-value	χ^2	d.o.f.
Diet	0.0476 *	9.6076	4
Habitat	0.8545	1.3403	4
Patch	0.4292	49.0906	48
Species	0.6540	117.2173	124
Species conservation status	0.0118 *	25.7054	12
Tipology	0.0200 *	45.4250	28

Table S4: Frequency anomalies for the regime classes as a function of the diet.

Diet	*Decline*	*Multiple boom–busts*	*Other*	*Rise*	*Single boom–bust*
GE	-2.3922	-3.4118	-0.2549	2.5882	3.4706
SP	2.3922	3.4118	0.2549	-2.5882	-3.4706

Table S5: Frequency anomalies for the regime classes as a function of the species ecological tipology.

Tipology	*Decline*	*Multiple boom–busts*	*Other*	*Rise*	*Single boom–bust*
duck	1.1078	0.0882	0.7451	-0.9118	-1.0294
flamingo	-0.2059	-0.4412	-0.0588	1.5588	-0.8529
grebe	-0.5490	2.8235	-0.1569	-1.1765	-0.9412
heron	-0.0980	-2.3529	-0.3137	1.6471	1.1176
larosterna	-1.2647	-0.8529	-0.6471	1.1471	1.6176
pelicaniform	-0.1373	-0.2941	-0.0392	1.7059	-1.2353
rail	1.5882	0.1176	-0.1176	-0.8824	-0.7059
wader	-0.4412	0.9118	0.5882	-3.0882	2.0294

Table S6: Frequency anomalies for the regime classes as a function of the species conservation status.

Status	*Decline*	*Multiple boom–busts*	*Other*	*Rise*	*Single boom–bust*
EN	-0.3431	1.2647	0.9020	-0.7353	-1.0882
LC	1.5000	0.5000	0.0000	-4.5000	2.5000
NT	0.2157	-1.8235	-0.5098	6.1765	-4.0588
VU	-1.3725	0.0588	-0.3922	-0.9412	2.6471

Table S7: Results of the Kruskal-Wallis tests for association between regimes and different patch- and species-related variables. Asterisks mark results below the threshold of 5% significance. For this test, species conservation status was converted into a numerical variable ranging from 1 to 5.

variable	p-value	H	d.o.f.
Generation time	0.0049 *	14.8872	4
Species conservation status	0.4166	3.9222	4
Weight	0.1725	6.3803	4

Table S8: Results of the chi2 tests for association between Canonical boom–busts and different patch- and species-related variables. Asterisks mark results below the threshold of 5% significance.

variable	p-value	χ^2	d.o.f.
Diet	1.0000	0.0000	1
Habitat	0.5832	0.3011	1
Patch	0.0169 *	24.5927	12
Species	0.4833	27.6470	28
Species conservation status	0.5833	1.9479	3
Tipology	0.0989	10.6765	6

Table S9: Frequency anomalies for the occurrence of Canonical boom–busts as a function of the patch.

Patch	Frequency anomaly
Delta del Millars	-0.1398
Hondo de Amorós	0.8602
Marjal de Almenara	2.3011
Marjal de Pego-Oliva	0.4409
Marjal de Xeresa-Xeraco	-0.4194
Marjal dels Moros	2.7419
P. N. Salinas de Santa Pola	-1.9570
P. N. de El Hondo	-2.2151
P. N. de L'Albufera	-0.5161
P. N. Lagunas de La Mata-Torrevieja	-0.2581
Prat de Cabanes-Torreblanca	-0.4194
Puerto de Castellón	-0.1398
Puerto de Valencia	-0.2796

Table S10: Results of the Kruskal-Wallis tests for association between Canonical Boom–Bust and different patch- and species-related variables. Asterisks mark results below the threshold of 5% significance. For this test, species conservation status was converted into a numerical variable ranging from 1 to 5.

variable	p-value	H	d.o.f.
Generation time	0.2616	1.2601	1
Species conservation status	0.9466	0.0045	1
Weight	0.3744	0.7890	1

Table S11: Correlations between patch total abundances and rainfall.

Patch	Pearson's r	p-value
Marjal de Almenara	-0.1187	0.4657
Marjal de Pego-Oliva	0.1553	0.3385
Marjal de Xeresa-Xeraco	-0.0222	0.8917
Marjal dels Moros	0.0484	0.7670
P. N. Salinas de Santa Pola	0.0457	0.7795
P. N. de El Hondo	-0.1130	0.4875
P. N. de L'Albufera	-0.0220	0.8930
P. N. Lagunas de La Mata-Torrevieja	0.1374	0.3978
Prat de Cabanes-Torreblanca	0.0088	0.9571
Puerto de Valencia	0.1561	0.3361
